# Comparative toxicity assessment of glyphosate and two commercial formulations in the planarian *Dugesia japonica*

**DOI:** 10.3389/ftox.2023.1200881

**Published:** 2023-06-26

**Authors:** S. Grace Fuselier, Danielle Ireland, Nicholas Fu, Christina Rabeler, Eva-Maria S. Collins

**Affiliations:** ^1^ Department of Biology, Swarthmore College, Swarthmore, PA, United States; ^2^ Department of Physics and Astronomy, Swarthmore College, Swarthmore, PA, United States; ^3^ Department of Neuroscience, Perelman School of Medicine, University of Pennsylvania, Philadelphia, PA, United States; ^4^ Center of Excellence in Environmental Toxicology, University of Pennsylvania, Philadelphia, PA, United States

**Keywords:** flatworm, glyphosate, new approach method, behavioral screening, neurotoxicity, GBH, herbicide, ecotoxicology

## Abstract

**Introduction:** Glyphosate is a widely used, non-selective herbicide. Glyphosate and glyphosate-based herbicides (GBHs) are considered safe for non-target organisms and environmentally benign at currently allowed environmental exposure levels. However, their increased use in recent years has triggered questions about possible adverse outcomes due to low dose chronic exposure in animals and humans. While the toxicity of GBHs has primarily been attributed to glyphosate, other largely unstudied components of GBHs may be inherently toxic or could act synergistically with glyphosate. Thus, comparative studies of glyphosate and GBHs are needed to parse out their respective toxicity.

**Methods:** We performed such a comparative screen using pure glyphosate and two popular GBHs at the same glyphosate acid equivalent concentrations in the freshwater planarian *Dugesia japonica*. This planarian has been shown to be a useful model for both ecotoxicology and neurotoxicity/developmental neurotoxicity studies. Effects on morphology and various behavioral readouts were obtained using an automated screening platform, with assessments on day 7 and day 12 of exposure. Adult and regenerating planarians were screened to allow for detection of developmentally selective effects.

**Results:** Both GBHs were more toxic than pure glyphosate. While pure glyphosate induced lethality at 1 mM and no other effects, both GBHs induced lethality at 316 μM and sublethal behavioral effects starting at 31.6 μM in adult planarians. These data suggest that glyphosate alone is not responsible for the observed toxicity of the GBHs. Because these two GBHs also include other active ingredients, namely diquat dibromide and pelargonic acid, respectively, we tested whether these compounds were responsible for the observed effects. Screening of the equivalent concentrations of pure diquat dibromide and pure pelargonic acid revealed that the toxicity of either GBH could not be explained by the active ingredients alone.

**Discussion:** Because all compounds induced toxicity at concentrations above allowed exposure levels, our data indicates that glyphosate/GBH exposure is not an ecotoxicological concern for *D. japonica* planarians. Developmentally selective effects were not observed for all compounds. Together, these data demonstrate the usefulness of high throughput screening in *D. japonica* planarians for assessing various types of toxicity, especially for comparative studies of several chemicals across different developmental stages.

## 1 Introduction

Glyphosate is a non-selective herbicide that inhibits the enzyme 5-enolpyruvylshikimate-3-phosphate (EPSP) synthase in plants. This enzyme catalyzes a key reaction in the shikimate pathway, which is responsible for the biosynthesis of three aromatic amino acids: phenylalanine, tyrosine, and tryptophan (Saunders and Pezeshki, 2015). Animals lack the shikimate pathway; thus, glyphosate has been deemed safe for humans and animals at recommended usage concentrations (reference dose of 0.3–1.75 mg/kg/day depending on the country/regulatory agency (Solomon, 2016)).

The growing use of genetically modified glyphosate-resistant crops has led to increased large-scale usage of GBHs (Duke, 2018), raising concerns about rising exposure levels. In the U.S., glyphosate usage increased from <4,000 tons in 1987 to >80,000 tons in 2007. It is estimated that by 2025 between 740,000—920,000 tons of glyphosate will be used globally each year (Maggi et al, 2020). Some epidemiological studies have found a concomitant increase in human exposure concentrations (Mills et al, 2017). This increasing glyphosate use raises concerns about possible adverse health effects in humans arising from low-dose chronic exposure and whether current exposure limits are sufficiently protective. These concerns can be partially attributed to conflicting regulatory guidelines. In 2017, the International Agency for Research on Cancer (IARC) concluded that glyphosate is “probably carcinogenic to humans” (IARC, 2017). In contrast, in February 2020, the U.S. Environmental Protection Agency (EPA) published its interim decision registration review for glyphosate, finding that glyphosate has “no risks of concern to human health” and is unlikely to be a human carcinogen at currently allowed levels (https://www.epa.gov/ingredients-used-pesticide-products/glyphosate). This decision has been challenged in court. The European Food Safety Authority (EFSA) is currently reviewing its approval of glyphosate, granting a 1-year extension until December 2023, to allow for sufficient peer review of existing data. Importantly, the types of studies considered by the different regulatory agencies can vary greatly and ultimately impacts their final decisions (Benbrook, 2019).

Animal testing and *in vitro* studies have found that exposure to glyphosate or GBHs can affect both adult and developing brain function, manifested as increased oxidative stress, mitochondrial dysfunction, and neuroinflammation (reviewed in (Costas-Ferreira et al, 2022; Lacroix and Kurrasch, 2023)). However, it remains unclear whether these effects occur at doses relevant to human exposure, especially as epidemiological studies have thus far only found weak or non-significant correlations between glyphosate exposure and health outcomes (Mink et al, 2011). Thus, although glyphosate may present a neurotoxic/developmentally neurotoxic hazard, it is unclear and controversial whether it constitutes an actual risk at current allowable exposure levels.

In common usage, glyphosate is used as part of a formulation. In commercial GBHs, glyphosate is used as an isopropylamine (most common), monoammonium, potassium, sodium, or trimesium salt to enhance its water solubility (Szekacs and Darvas, 2012; IARC, 2017). Glyphosate (acid equivalent [ae]) concentrations range from less than 1% to >70% (w/w), depending on the GBH product (IARC, 2017). Some commercial glyphosate formulations also include other active ingredients (e.g., diquat dibromide), particularly because of the growing concern of glyphosate-resistant weeds (Nandula et al, 2005; IARC, 2017). Commercial GBHs also contain various additives and surfactants (e.g., polyoxyethylene amine (POEA)), to enhance absorption into plants (Riechers et al, 1995; Szekacs and Darvas, 2012). Despite being largely disregarded in the safety assessments for GBHs, some animal and human cell culture studies have shown that these adjuvants may have their own intrinsic toxicity or could have synergistic effects to enhance the toxicity of glyphosate (Mesnage et al, 2019; Defarge et al, 2018). The exact composition of a product is considered proprietary and thus the identity and concentrations of non-active ingredients are largely unknown, making comparative studies of individual ingredients difficult. Thus, it is important to understand the toxicity of both commercial GBHs and of pure glyphosate.

Here, we use an aquatic invertebrate model, the freshwater planarian *Dugesia japonica*, to evaluate the toxicity of pure glyphosate and GBHs. This model has been shown to be well-suited for both ecotoxicity (Wu and Li, 2018) and developmental neurotoxicity (Hagstrom et al, 2016; Ireland and Collins, 2022; 2023) studies, thus allowing for evaluation of both aspects simultaneously. Because of their regenerative capabilities and similar size, both adult and developing planarians can be assessed in parallel using the same assays. This uniquely allows for a direct comparison of neurotoxic *versus* developmental neurotoxic effects. Understanding potential effects on different developmental stages, even at non-environmentally relevant concentrations, can provide insight on the mechanisms of neurotoxicity (Ireland et al, 2022).

Multiple studies have shown that planarians are sensitive to GBH exposure (Córdova López et al, 2019; Sheehan and Hoegler, 2018; Zhang et al, 2020; 2018; 2023). Studies with the species *D. japonica* found that exposure to sublethal concentrations (32–96 mg/L) of glyphosate (ae) for 1–5 days affected various biomarkers of oxidative stress, including catalase and glutathione S-transferase (GST) expression and activity (Zhang et al, 2018; 2020). Exposure to 64 mg/L glyphosate (ae) for up to 5 days was found to cause morphological damage, such as abnormal body shapes, tissue damage, and significant changes to gene expression (Zhang et al, 2023). Studies with the planarian species *Girardia tigrina* found that planarian regeneration and behavior (locomotion, feeding, and phototaxis) were altered by 4 day GBH exposure at concentrations as low as 3.75 mg/L (Sheehan and Hoegler, 2018; Córdova López et al, 2019). However, a major limitation with existing studies is that they only considered one GBH, which differed across the studies. Thus, it is unknown if the observed toxicity was due to glyphosate or other GBH components.

We tested pure glyphosate and two GBHs which are popular and commercially available in the United States: Roundup^®^ Concentrate Plus ((RC); glyphosate isopropyl amine salt concentration: 18% of total product) and Roundup^®^ Ready-to-Use ((RR); glyphosate isopropyl amine salt concentration: 2% of total product). Both formulations also list a second active ingredient (0.73% diquat dibromide (RC); 2% pelargonic acid and related fatty acids (RR)). We compared the toxicity of the two GBHs and pure glyphosate at fixed concentrations of glyphosate (ae), spanning 1 μM −1 mM*.* These concentrations were chosen to encompass environmentally relevant concentrations and concentrations previously shown to induce toxicological outcomes in planarians. We also investigated the toxicity of the other active ingredients (diquat dibromide and pelargonic acid) at the equivalent concentrations tested in their respective GBHs. Through this direct comparison, we can determine whether glyphosate alone, the other active ingredients, or the mixture are harmful to planarians at typical environmental levels. In addition, we can evaluate whether these products—at any concentration—affect brain development and function by comparing the toxic effects seen in adult *versus* regenerating planarians.

Toxicity was assessed using high-throughput screening (HTS) in 96-well plates by evaluating lethality, morphology, and several behavioral endpoints on days 7 and 12 of exposure using an automated screening platform (Zhang et al, 2019). Both adult and regenerating planarians were screened to allow for identification of any developmentally selective effects. We also measured the effect of glyphosate on acetylcholinesterase (AChE) activity and on GST activity. We found that both GBHs were more toxic than equivalent concentrations of pure glyphosate. Because adverse effects were only found at high concentrations, our results suggest that neither glyphosate nor the tested GBHs pose an ecotoxicological risk to *D. japonica* at expected environmental concentrations.

## 2 Materials and methods

### 2.1 Test animals

Freshwater planarians of the species *D. japonica* that have been maintained in the laboratory for over a decade were primarily used. To compare species differences, *Dugesia dorotocephala* and *G. tigrina* planarians (both listed as “brown planarians”, Carolina Biological Supply, Burlington, NC) were used in some bulk lethality experiments. All planarians were stored in 1x Instant Ocean (IO, Blacksburg, VA) in Tupperware containers and kept at 18°C in a Panasonic refrigerated incubator in the dark. The animals were fed organic beef liver (obtained from a local farm) or chicken liver (Bell and Evans, Fredericksburg, PA) once a week. The containers were cleaned twice a week following standard protocols (Dunkel et al, 2011). Fully regenerated worms starved ≥5 days and gliding normally in the container were used for all experiments. Planarians were selected to fall within a certain range of sizes, with larger planarians used for amputation/regeneration experiments. Thus, the final sizes of adult planarians and regenerating tails within a given experiment were similar (HTS: ∼3–6 mm, bulk: ∼5–8 mm, biochemical assays: ∼8–12 mm). To induce regeneration/development, intact planarians were amputated on day 1 via a transverse cut between the auricles and the pharynx with an ethanol-sterilized razor blade. Chemical exposure began within 3 h of amputation.

### 2.2 Chemical preparation

Pure glyphosate (CAS # 1071-83–6) was purchased from Sigma-Aldrich (St. Louis, MO) and had a purity ≥98%. Diquat dibromide (CAS # 6385-62–2, analytical standard) and Pelargonic acid (CAS # 112-05–0, purity 97.5%) were purchased from ChemService (West Chester, PA). Two formulations of Roundup^®^ were tested: Roundup^®^ Concentrate Plus (RC, product number LB5778, active ingredients: 18% glyphosate isopropyl amine salt, 0.73% diquat dibromide) and Roundup^®^ Ready-to-Use Weed and Grass Killer III (RR, product number LB5135, active ingredients: 2% glyphosate isopropyl amine salt, 2% pelargonic and other related fatty acids). The original commercial stocks were stored at room temperature in the dark. To compare equivalent concentrations of the free acid form of glyphosate to the salt forms found in the GBHs, the acid equivalent (ae) concentrations of glyphosate in each formulation were used. Thus, glyphosate and both Roundup^®^ formulations were compared at equivalent concentrations of glyphosate (ae, 1, 3.16, 10, 31.6, 100, 316 and 1,000 μM, Table 1). Diquat dibromide was tested at the equivalent concentrations found in the tested concentrations of RC, which corresponded to 0.0092, 0.029, 0.092, 0.29, 0.92, 2.9, and 9.2 mg/L. Pelargonic acid was tested at the equivalent concentrations found in the tested concentrations of RR, which corresponded to 0.282, 0.892, 2.82, 8.92, 28.2, 89.2, and 282 mg/L. In the HTS experiments, 100 μM L-ascorbic acid (CAS # 50-81–7, Sigma-Aldrich) was assayed as a negative control. Stocks of 10X the highest tested concentration of each test compound were prepared fresh in IO water on the day of experimental set-up.

**TABLE 1:** Glyphosate (ae) concentrations tested.

Concentration (µM)	Concentration (mg/L, ppm)
1	0.169
3.16	0.534
10	1.69
31.6	5.34
100	16.9
316	53.4
1,000	169

### 2.3 High-throughput screening (HTS)

#### 2.3.1 Plate setup and screening

HTS was conducted in tissue culture-treated 96-well plates (Genesee Scientific, San Diego, CA). Each 96-well plate contained 7 chemical concentrations and one in-plate IO water (solvent) control, with one concentration per row, and each row containing 12 planarians. Each well contained one adult planarian or one regenerating tail piece in 200 µL of the nominal concentration of test solution. The plates were sealed with a ThermalSeal RTS seal (Excel Scientific, Victorville, CA) (Zhang et al, 2019). Experiments were performed in duplicate for a total of n = 24 per concentration. The orientation of the concentrations in the plate was shifted down 3 rows in the second replicate to control for edge effects (Zhang et al, 2019). For each chemical and experiment, one plate containing adult (intact) planarians and one plate containing regenerating tails (2 plates total) were assayed. The plates were stored in stacks in the dark at room temperature when not being screened.

Screening was performed on an automated platform, described extensively in (Zhang et al, 2019; Ireland et al, 2020; 2022). Outcome measures were obtained from studying planarian morphology and behavior on the assay stations (phototaxis/locomotion/morphology, stickiness, and noxious heat sensing/scrunching) and analyzed using the same pipeline as in (Zhang et al, 2019; Ireland et al, 2020; 2022). The parameters of each assay were updated from previous iterations to be optimized for 96-well plates. Briefly, on day 7 and day 12, phototaxis and stickiness were assayed. Phototaxis was performed as follows: 1-min red light (first dark cycle), 2-min blue light (light cycle), 2-min red light (second dark cycle) and imaged with a high-resolution camera (Basler acA5472, Basler, Germany). Data from the phototaxis assay was used to manually score lethality and abnormal body shapes. Dead planarians were excluded from all further analysis. Various measures of locomotion, both with and without light stimulus, were also obtained from the phototaxis assay. Wall preference/anxiety scores (Bayingana et al, 2022; Ireland et al, 2022), average gliding speed and percent time resting were calculated during the second dark cycle, with speeds <0.2 mm/s considered resting. The total number of locomotor bursts in the entire phototaxis assay were also calculated (Ireland et al, 2022). To score whether a planarian showed a reaction to the blue light stimulus, the average speed in the second minute of the light cycle and in the first dark cycle were calculated. Average speeds <0.1 mm/s were set to 0.1 mm/s to reduce background noise in nonmoving worms. The ratio of the speed in the second minute of the light cycle/the speed in the first dark cycle was calculated. If this ratio exceeded 1.1 (i.e., a 10% increase in speed during the light cycle), the planarian was scored as successfully phototaxing. Stickiness measures the number of planarians that are stuck/unstuck when the plate is shaken at a fixed rotation per minute (RPM) (Ireland et al, 2020). The RPM settings were 1097 RPM for adult plates on day 7, 1200 RPM for regenerating plates on day 7, and 1350 RPM for all plates on day 12. Stickiness was assessed both before (A) and after (Z) the phototaxis assay. Additionally, on day 12, scrunching/noxious heat sensation was assayed. The plate was placed on a peltier plate (TE Technology Inc., Traverse City, MI) to gradually heat up the water in the wells. Between plates, the peltier was set to 43°C. At the start of the assay, the temperature of the peltier was initially set to 65°C. After 5.5 min, the peltier was switched to 54°C for the remaining 3 min of the assay to stabilize the water temperature. From this assay, the planarians’ ability to scrunch (Cochet-Escartin et al, 2015), and the dynamics of their response to heat (rate and strength of reaction (Ireland et al, 2020)) were measured. Screening data was analyzed using MATLAB (MathWorks, Natick, MA). Data analysis was performed blinded with no chemical information provided. The data from both runs were compiled for a total dataset of n = 24 per chemical condition.

#### 2.3.2 Statistical analysis

Statistical analysis was performed in R (version 4.1.2 (R Core Team, 2016)) for each chemical, worm type (adult or regenerating) and day separately. For binary endpoints, (lethality, stickiness, phototaxis, scrunching, and body shape), a Fisher’s exact test comparing each concentration to the respective in-plate control population was performed. *p*-value adjustments were made using the Benjamini and Hochberg method for correction for multiple testing (Benjamini and Hochberg, 1995). For continuous endpoints, the distribution of the data was first visually inspected for normality. Normal endpoints (speed, locomotor bursts, noxious stimuli rate and noxious stimuli strength), were evaluated using a one-way Welch’s ANOVA to test for the effect of concentration, followed by a *post hoc* Tamhane-Dunnett test using the package PMCMRplus. For endpoints with a skewed distribution (resting and anxiety), a Kruskall-Wallis omnibus test was performed followed by a *post hoc* Dunn test with Benjamini and Hochberg *p*-value adjustment using the PMCMRplus package. For all statistical tests, comparisons were made with the respective in-plate IO controls and adjusted *p*-values less than 0.05 were considered significant. For all non-lethality endpoints, concentrations with statistically significant lethality were removed before statistical testing and not considered. Statistical tests were either one- or two-tailed depending on the expected direction of effects. A summary of the statistical methods for each endpoint is provided in Supplementary Table S1. Furthermore, to decrease false positives due to plate-to-plate variability, we calculated “biological relevancy cutoffs” (Zhang et al, 2019) as either the mean +/- 2 standard deviations or the 5th −95th percentiles of the compiled scores of the IO controls for each endpoint, depending on the normality of the distribution. To this end, we screened an additional plate of controls exposed to IO water alone to increase the number of control populations. Thus, in total we had 10 controls populations each with n = 24, per worm type. Statistically significant hits that fell within the biological relevancy cutoffs were excluded. Hits which were inconsistent across the two runs were also discarded. Potency was quantified as lowest-observed-effect-levels (LOELs), i.e., the lowest concentration with a statistically significant effect. For simplicity, only concentration-dependent hits are discussed in the Results, but all compiled data are available in Supplementary File S1. All R codes used in this manuscript are available at https://github.com/Collinslab-swat/Planarian-glyphosate.

### 2.4 Bulk lethality experiments

To test whether lethality was affected with different exposure set-ups and for possible species differences in susceptibility (Ireland et al, 2020), *D. japonica, G. tigrina* and *D. dorotocephala* planarians were exposed in bulk to RR or RC at concentrations corresponding to 31.6, 100, or 316 µM glyphosate ae or to IO water (solvent control). These concentrations were chosen to cover sublethal and lethal concentrations seen in the HTS experiments with *D. japonica*. *D. japonica* planarians were also exposed to 100, 316, and 1,000 µM glyphosate and 1,000 µM RR or RC. Planarians were exposed in tissue culture-treated 12-well plates (Genesee Scientific). For *D. japonica* and *G. tigrina* experiments, 3 wells each containing 6 planarians in 1.2 mL of the test solution were tested. For *D. dorotocephala* experiments, 4 wells each containing 6 planarians in 1.2 mL of test solution were tested. Thus, in all tests the ratio of chemical/planarian (200 µL/planarian) was consistent with HTS. Lethality was manually scored at day 2, day 4, day 8, and day 10. The *D. japonica* experiments were also checked on day 7 and day 12 for comparison to HTS. To statistically evaluate whether exposure type (96-well or bulk exposure) had a significant effect on *D. japonica* lethality, a generalized loglinear model was created testing the interactions of lethality:concentration:worm type (adult or regenerating): exposure type. The statistical significance of each term was determined by dropping each term and testing the significance compared to the original model using a one-way ANOVA. *p*-values for these comparisons are provided in Supplementary Table S2. The same approach was used to evaluate the effect of species by testing the interactions of lethality:concentration:worm type (adult or regenerating):species. *p*-values for the species comparison are provided in Supplementary Table S3.

### 2.5 Biochemical assays

#### 2.5.1 Exposure

For each experiment, at least 30 adult planarians were exposed to either IO water or 100 µM of either glyphosate, RR, or RC. This concentration was chosen as it was the highest concentration that was sublethal for all three chemical formulations. As an assay positive control for AChE inhibition, planarians were exposed to either 0.178 µM diazinon (CAS # 333-41–5, Sigma-Aldrich) in 0.5% dimethyl sulfoxide (DMSO, Sigma-Aldrich) or to 0.5% DMSO (solvent control). This concentration of diazinon has robustly caused significant planarian AChE inhibition in our laboratory (Ireland et al, 2022). Planarians were exposed in tissue culture-treated 12-well plates, with 6 planarians in 1.2 mL of the test solution per well, thus keeping the ratio of chemical/planarian consistent with the HTS set-up. Any fission events or planarians from wells with death were excluded from the assay.

#### 2.5.2 Acetylcholinesterase (AChE) activity assays

After exposure, the planarians were washed 3X with IO water and then homogenized in 200 µL 1% Triton X-100 in PBS as described in (Hagstrom et al, 2017; 2018). Briefly, after sitting on ice for about 15–20 min, the homogenate was centrifuged at 20,817 x *g* at 4°C for 30 min. The supernatant (clarified homogenate) was transferred to a clean, chilled tube and subsequently used. An Ellman assay (Ellman et al, 1961) was performed on the clarified homogenate using an Acetylcholinesterase Activity Assay kit (Sigma-Aldrich). Absorbance was read at 412 nm every minute for 10 min using a SpectraMax ABS Plus (Molecular Devices, San Jose, CA) spectrophotometer. AChE activity was calculated as the rate of change of absorbance per minute during the linear portion of the reaction. AChE activity was normalized by protein concentration as determined by a Coomassie (Bradford) protein assay kit (Thermo Scientific, Waltham, MA) and compared to the average respective solvent control samples (set at 100% activity) tested on the same day. Activity measurements were performed with 3 technical replicates per condition and 4 independent experiments (biological replicates).

#### 2.5.3 Glutathione S-transferase (GST) activity assays

After exposure, the planarians were washed 3X with IO water, flash frozen, and stored at −80°C. On the day of the experiment, the planarians were homogenized in 200 µL cold PBS similar to as done for the Ellman assay. After sitting on ice for about 10 min, the homogenate was centrifuged at 20,817 x *g* at 4°C for 30 min. The supernatant was transferred to a clean, chilled tube and subsequently used. The protein concentration of each homogenate was determined using a Coomassie (Bradford) protein assay kit. The GST activity of each sample was determined using a Glutathione-S-Transferase Assay kit (Sigma-Aldrich), using 12 µL of each homogenate, normalized to 4 mg/mL protein, in a 96-well plate. After addition of the substrate solution, absorbance was read at 340 nm every minute for 10 min using a SpectraMax ABS Plus (Molecular Devices, San Jose, CA) spectrophotometer. GST activity was calculated as the rate of change of absorbance per minute during the linear portion of the reaction and compared to the average of the IO water control samples (set as 100% activity) tested on the same day. Activity measurements were performed with 3 technical replicates per condition and 4 independent experiments (biological replicates).

#### 2.5.4 Statistical analysis

Statistical analysis of biochemical assays was performed in MATLAB. First, the distributions of each population (n = 4) were confirmed to be normal using Lilliefors test (lillietest) and visually inspected for equal variance. Then a one-way ANOVA was performed. If the means of the groups were found to be statistically different (*p* < 0.05), pairwise comparisons against the control was performed using Dunnett’s test.

## 3 Results

### 3.1 GBHs caused greater lethality than glyphosate

**FIGURE 1:**
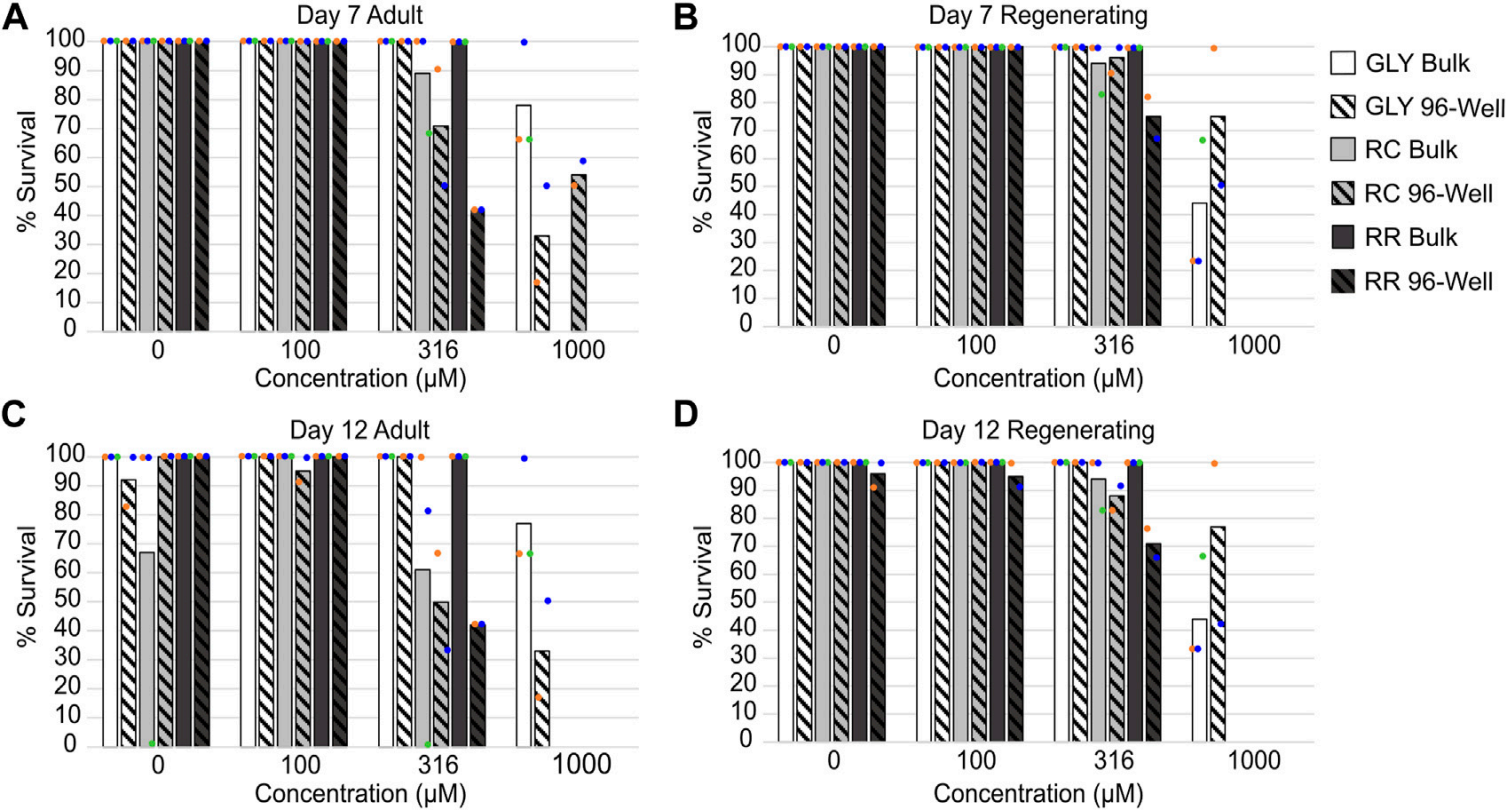
Comparison of lethality in different exposure conditions. **(A, B)** Percent survival at day 7 for **(A)** adult and **(B)** regenerating planarians**. (C, D)** Percent survival at day 12 in **(C)** adult planarians and **(D)** regenerating planarians. Concentrations refer to glyphosate acid equivalent concentrations. Bars indicate the average of all replicates (colored dots; 2 for 96-well plates and 3 for bulk).

All tested formulations caused significant lethality to both adult and regenerating *D. japonica* planarians at 1 mM on both days 7 and 12 when exposed in 96-well plates. Significant lethality was observed at lower concentrations for the two GBHs than for glyphosate alone. Significant lethality in both adult and regenerating planarians was induced by 316 μM RR, while 316 µM RC only caused significant lethality in adult planarians (Figure 1). As previous studies with planarians have primarily used bulk exposure, we compared the lethal effects of glyphosate, RR and RC when exposing single planarians in 96-well plates or 6 planarians/well in 12 well plates (Figure 1). We found a slight increase in lethal effects when using 96-well plates, with the exception of glyphosate exposure in regenerating planarians, which caused greater lethality in bulk exposure *versus* 96-well plates at 1 mM. The significance of these observations were quantified by analyzing the interaction between lethality, concentration, worm type (adult or regenerating) and exposure type (96-well or bulk) using a generalized linear model. At both day 7 and day 12, exposure type and worm type had a significant effect on lethality for glyphosate and RC (Supplementary Table S2). Bulk exposure also showed greater variability across the independent wells/replicates (individual dots in Figure 1) than the 96-well plate data.

We also compared the lethal effects of RR and RC on two other planarian species, *D. dorotocephala* and *G. tigrina*, using bulk exposure in 12-well plates. *D. dorotocephala* and *G. tigrina* were more sensitive than *D. japonica* to both RR and RC as 316 µM of either GBH caused at least 50% lethality by day 10 in both developmental stages (adult and regenerating), though the temporal dynamics differed (Supplementary Figure S1). Analysis of the interaction between lethality, concentration, worm type (adult or regenerating) and species using a generalized linear model revealed that species and worm type had a significant effect on lethality for both RR and RC.

### 3.2 RR and RC, but not glyphosate, show sublethal behavioral effects in adult planarians

**FIGURE 2:**
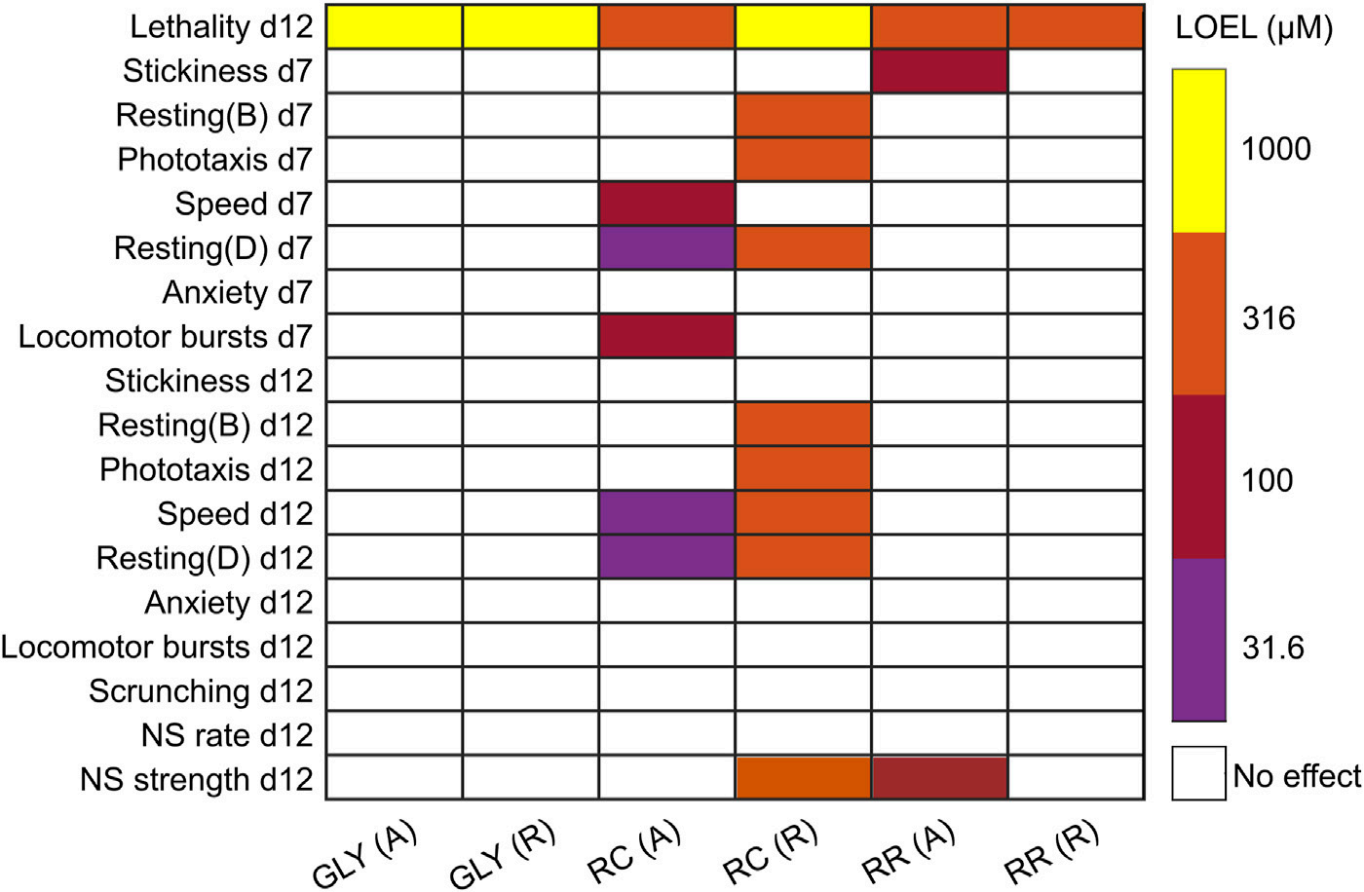
Comparison of toxicity of glyphosate, RR and RC exposure in adult and regenerating planarians. Heatmap comparing the lowest observed effect levels (LOELs) for glyphosate (GLY), RR, and RC exposure in adult (A) and regenerating (R) planarians in all tested endpoints. Percent time resting was calculated in both the dark (D) and blue (B) light periods of the phototaxis assay. NS: Noxious stimuli. Only concentration-dependent hits are shown. For simplicity, only the results from stickiness (Z) are shown as no significant hits were found in stickiness (A). The negative control, 100 μM L-ascorbic acid, showed no significant effects in any of the endpoints. All compiled data can be found in Supplementary File S1.

**FIGURE 3:**
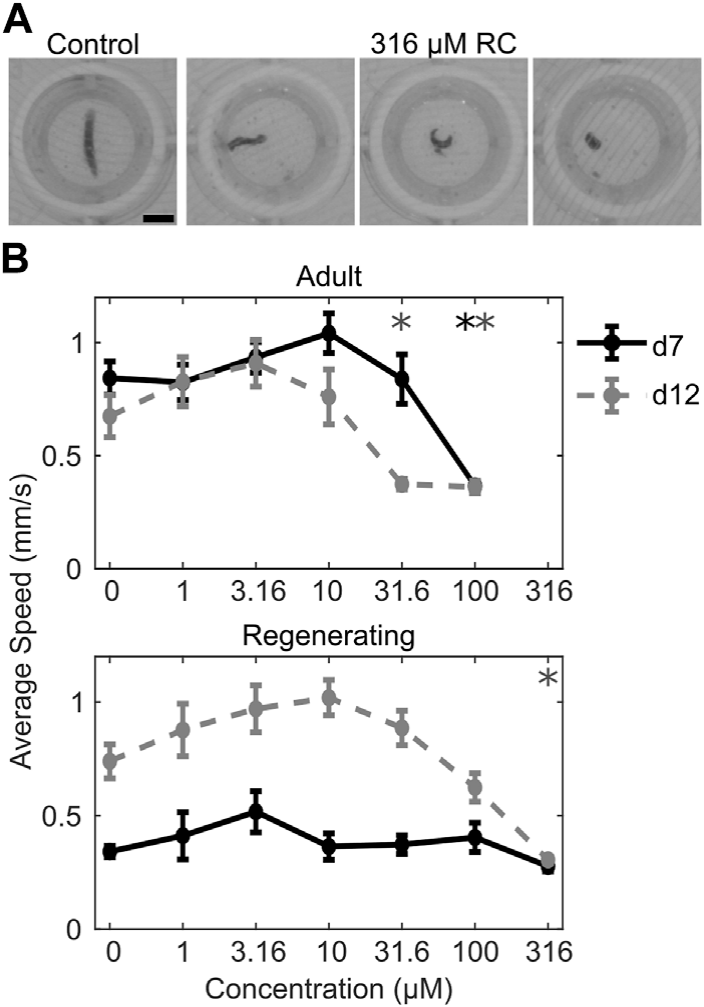
Sublethal effects of RC exposure. **(A)** Example images of a normal control planarian and the abnormal body shapes observed with 316 µM RC exposure in regenerating planarians. Abnormal body shapes (from left to right) are corkscrew/twisted, C-shape, contracted. Scale bar: 2 mm. **(B)** Concentration-response curves for adult (top) and regenerating (bottom) planarians exposed to RC. Symbols represent the mean of the speed in the dark during the phototaxis assay of n = 24 planarians. Error bars are the standard error. Speed was evaluated on both day 7 (d7, black solid line) and day 12 (d12, gray dashed line). 316 μM RC was lethal to adult planarians and thus no data are shown for adult planarians at this concentration. *p < 0.05 using a Welch’s ANOVA test followed by a pairwise Tamhane-Dunnett test comparing to the respective in-plate control population.

Morphological and behavioral defects at sublethal concentrations were assessed using an automated platform that evaluates morphology and various behaviors. Screening was done on both adult and regenerating planarians to assess whether differential sensitivity was present in the two developmental stages. Assessments were done on day 7 and day 12 to evaluate the dynamics of effects. A comparison of the LOEL for each formulation and endpoint is presented in Figure 2. Glyphosate did not induce any significant effects at sublethal concentrations. RR induced specific effects in stickiness at day 7 and in noxious stimuli strength at day 12 at 100 µM. Notably, these effects were only seen in adult planarians, whereas regenerating planarians showed no sublethal effects of RR exposure. RC exposure induced significant sublethal behavioral effects. Although 316 µM RC was not significantly lethal to regenerating planarians as it was in adult planarians, this concentration caused severe toxicity in the regenerating planarians. Regenerating planarians exposed to 316 µM RC showed abnormal body shapes including corkscrews, C-shapes, and contraction on both day 7 and day 12 (Figure 3A). On day 7 and day 12, 78% (n = 23; *p* = 7.4 × 10^−9^, Fisher’s Exact Test with BH correction) and 67% (n = 21; *p* = 7.0 × 10^−7^, Fisher’s Exact Test with BH correction) of regenerating planarians exposed to 316 µM RC showed any abnormal body shape, respectively. In-plate controls and all lower concentrations did not have any planarians with abnormal body shapes. These abnormal shapes were correlated with locomotion defects, including reduced speed (Figure 3B), increased resting, and defects in phototaxis and the noxious heat response. Together, these suggest that 316 µM RC caused systemic toxicity in regenerating planarians. Adult planarians showed greater sensitivity to RC exposure, with significant sublethal effects on motility/locomotion, manifested as decreased speed and increased resting, starting at 31.6 µM (Figure 3B).

### 3.3 Effects on biomarkers

**FIGURE 4:**
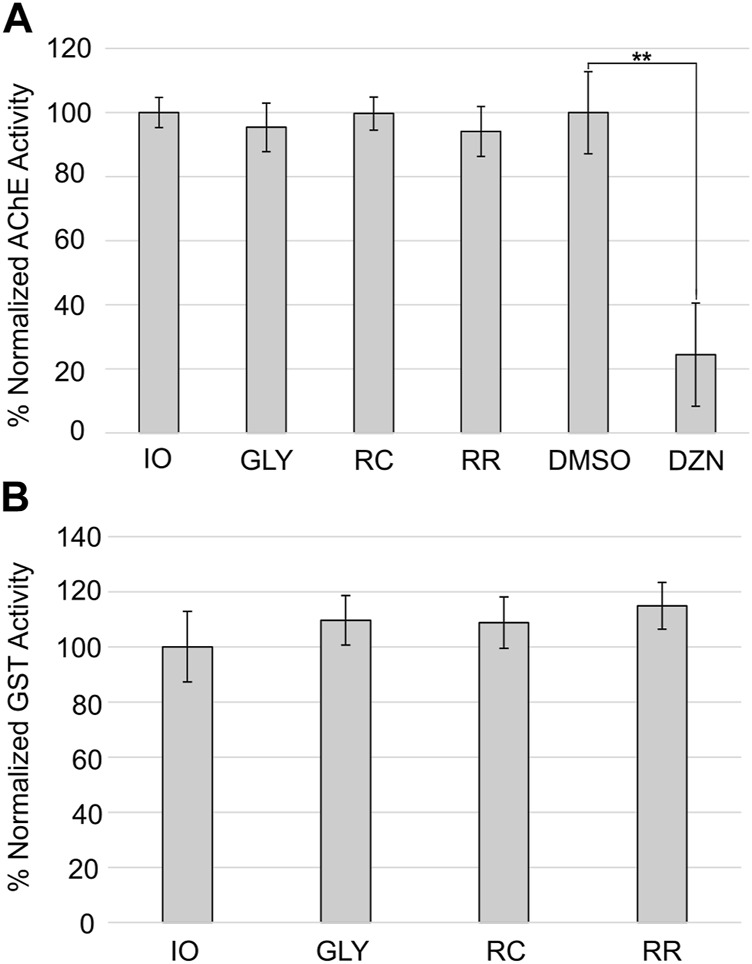
Effects of glyphosate and GBHs on biomarkers. **(A)** Percent normalized AChE activity in adult planarians after 12-day exposure to 100 µM glyphosate (GLY), RC, or RR. Exposure to 0.178 µM diazinon (DZN) was compared to its vehicle control 0.5% DMSO as a positive control for AChE inhibition. **(B)** Percent normalized GST activity in adult planarians exposed to 100 µM GLY, RC, or RR for 12 days. **p < 0.01 using a one-way ANOVA followed by a *post hoc* Dunnett test compared to the respective control population. Data shown as the mean of 4 biological replicates. Error bars represent the standard deviation.

We tested whether 12-day exposure to the highest tested sublethal concentration (100 µM) glyphosate, RR, or RC affected AChE activity in adult planarians (Figure 4A). Only the positive control diazinon showed a statistically significant decrease in AChE activity (one-way ANOVA, followed by Dunnett’s test, *p*<<0.01). None of the GBHs had a statistically significant effect on AChE activity.

Many studies have suggested that glyphosate and GBHs can cause oxidative stress (reviewed in (Costas-Ferreira et al, 2022)). Thus, we evaluated the effects of glyphosate/GBH exposure on the activity of a common biomarker of oxidative stress—GST—at the highest concentration of glyphosate (ae) that was non-lethal for all three compounds (i.e., 100 µM). GST has been previously characterized in *D. japonica* (Zhang et al, 2020). We found no significant effects using a one-way ANOVA on GST activity with any of the tested compounds at 100 µM in adult planarians after 12 days of exposure (Figure 4B).

### 3.4 Diquat dibromide shows similar toxicity to RC

RC and RR also contain other active ingredients besides glyphosate (diquat dibromide and pelargonic acid, respectively), that may contribute to the toxicity of the GBHs. Thus, we screened diquat dibromide and pelargonic acid alone at the equivalent concentrations that would be found in the tested concentrations of the respective GBHs. Pelargonic acid did not show any significant effects in any endpoints, suggesting no toxicity to planarians up to 282 mg/L. In contrast, diquat dibromide caused lethality at day 12 at the highest tested concentration (9.2 mg/L, equivalent to the concentration of diquat dibromide found in RC at 1,000 µM glyphosate ae) in adult planarians. Diquat dibromide also caused many sublethal behavioral effects across different endpoints, starting at 0.29 mg/L (Supplementary Figure S2A). For adult planarians, the overall LOEL for diquat dibromide was equivalent to the LOEL found for RC (RC at 31.6 µM glyphosate ae contains 0.29 mg/L diquat dibromide), though the toxicological profiles (exact endpoints affected) differed slightly. Regenerating planarians exposed to diquat also demonstrated abnormal behavior starting at 2.9 mg/L, though no significant lethality was induced up to 9.2 mg/L. As in RC, exposure to diquat induced abnormal body shapes. However, unlike RC, these were primarily found in adult planarians and were highly dynamic as the planarians writhed uncontrollably in C- and S-like shapes (Supplementary Figure S2B). In adult planarians, abnormal body shapes were induced starting at 0.92 mg/L on both day 7 and day 12, whereas regenerating planarians only demonstrated abnormal body shapes on day 12 at 9.2 mg/L (Supplementary Figure S2C).

## 4 Discussion

### 4.1 GBHs showed greater toxicity than pure glyphosate in *Dugesia japonica*

We compared the toxicity of glyphosate alone to that of two common GBHs in freshwater planarians. Both GBHs were more toxic than pure glyphosate, which only showed effects in lethality at the highest test concentration (1 mM). Regarding lethality, RR was the most toxic, with lethal effects in both adult and regenerating planarians at 316 µM. RC was slightly less toxic than RR, as significant lethality was not observed at 316 µM RC in regenerating planarians. However, these planarians displayed obvious signs of toxicity (abnormal body shapes, immobility). RC had an overall LOEL of 31.6 µM in adult planarians and caused much stronger sublethal phenotypic effects than RR as effects on multiple locomotion-based endpoints were observed. In contrast, RR only caused increased stickiness and a more sensitive response to noxious heat (NS strength endpoint) only in adult planarians at 100 µM. We have previously found that inhibition of AChE activity can correlate with effects in these endpoints (Ireland et al, 2022). As an organophosphonate, glyphosate is a weak inhibitor of mammalian AChE (Larsen et al, 2016). However, neither glyphosate nor the GBHs had a statistically significant effect on AChE activity at sublethal concentrations (100 µM), suggesting AChE inhibition is not the cause of the observed behavioral effects. None of the test formulations had a significant effect on GST activity at 100 μM, suggesting that the observed behavioral phenotypes are not correlated with oxidative stress.

Previous work found that *D. japonica* exposed to 32 mg/L (approximately 189 µM) glyphosate ae for 3 or 5 days had altered GST expression and activity (Zhang et al, 2020), though the directionality of the effects on GST activity differed depending on the day tested. On day 3, increased GST expression and activity was observed compared to controls, whereas on day 5 GST expression was indistinguishable from controls and decreased activity was observed. In agreement with our findings, 96 h exposure in developing zebrafish to Roundup^®^ Ultramax did not affect oxidative stress biomarkers even at concentrations that induced lethality and developmental effects (Lanzarin et al, 2019). Effects on gene expression of several oxidative stress-related markers was upregulated in *Caenorhabditis elegans* following 24 h glyphosate exposure; however, only at concentrations above the lethality LOEL (García-Espiñeira et al, 2018). Thus, although oxidative stress has been suggested to be connected to the potential neurotoxic effects of GBHs (Costas-Ferreira et al, 2022), these effects may only be relevant at high concentrations which non-specifically induce systemic toxicity/lethality.

The observation that the GBHs were more toxic than pure glyphosate suggests that the additional components of the GBHs either enhance the toxicity of glyphosate or are toxic on their own. Recent work has found that several GBHs (including RC), but not glyphosate up to 10 mM, were cytotoxic and genotoxic in human cell culture (Smith-Roe et al, 2023). This agrees with a similar study comparing the cytotoxicity of 14 different GBHs and pure glyphosate in human embryonic kidney cells. This work found that the cytotoxicity of the GBHs (1/LC_50_ [concentration that induced 50% lethality]) could be up to 358 times greater than pure glyphosate, though large variability was also seen across the different formulations (Defarge et al, 2018).

As there are over 750 different GBHs sold in the U.S. (Henderson et al, 2010), with varying—and largely unknown—formulations, it can be difficult to dissect which specific co-formulants may drive GBH toxicity. In this study, the observed toxicity of RC in planarians may be partially attributed to the other active ingredient, diquat dibromide, a non-selective herbicide and desiccant. We found that diquat had strong effects on planarian body shapes and locomotion, especially in adult planarians. In human cell genotoxicity tests, diquat dibromide alone was cytotoxic, genotoxic, and clastogenic, likely due to its effects on oxidative stress. However, RC was not found to be genotoxic (Smith-Roe et al, 2023). In contrast, the U.S. EPA has classified diquat dibromide as “not likely to be carcinogenic” due to lack of effects seen in *vivo* animal studies (USEPA United States Environmental Protection Agency, 1995). Together, these suggest that diquat dibromide may not be genotoxic *in vivo* or at the low concentrations (0.73%) used in RC. Diquat dibromide has been shown to be lethal to a variety of aquatic organisms with LC_50_ values ranging from ∼1 to 300 mg/L (Wilson and Wu, 2012). We estimate that the concentration of RC that was lethal to adult planarians (316 µM) contained ∼2.9 mg/L diquat dibromide. Diquat alone only induced statistically significant lethality (29%) starting at 9.2 mg/L in adult planarians at day 12. However, sublethal concentrations of diquat induced a large range of locomotor and morphological defects that differed slightly from the toxicological profile of RC, though the LOELs were equivalent (RC at 31.6 µM glyphosate ae contains 0.29 mg/L diquat). Future work could explore whether the binary mixture of glyphosate and diquat dibromide could recapitulate the toxicity profile of RC or whether interactions with other adjuvants may also play a role, to better understand the toxicity mechanisms. For practical purposes, it is important to keep in mind that these concentrations are all well above the maximum expected environmental concentration (0.0031 mg/L) (Wilson and Wu, 2012).

RR contains a different active ingredient beside glyphosate: “pelargonic acid and related fatty acids”. Pelargonic acid, also called nonanoic acid, is a naturally occurring nine-carbon fatty acid that is used as an herbicide. Pelargonic acid is considered to have little to no risk for mammalian toxicity and is even approved as a food additive in the U.S. While mild toxicity has been observed with pelargonic acid in zebrafish (Techer et al, 2015), we observed no significant effects with up to 282 mg/L pelargonic acid alone. Thus, these data suggest that the toxicity of RR cannot be explained by the toxicity of its active ingredients (glyphosate and pelargonic acid) when tested individually. However, it is possible that synergistic effects of the different components enhance their toxicity.

GBHs also contain other additives such as surfactants or contaminants such as heavy metals, which have been suggested to have their own toxicity, especially to aquatic life (Defarge et al, 2018). For example, the surfactant polyoxyethylene 15 (POE 15) tallow amine and a GBH containing it were found to be 10- to 40-times more toxic than pure glyphosate to a variety of aquatic invertebrate species (Folmar et al, 1979), with the toxicity of the surfactant alone sharing many similarities to that of the GBH. Similar increases in toxicity of POE 15 over glyphosate have also been found in regulatory animal tests (Mesnage et al, 2019) and in human cell culture studies (Defarge et al, 2018). GBHs containing POEAs were also found to be correlated with more severe symptoms of acute toxicity in human poisoning cases than GBHs without POEAs (Langrand et al, 2020). The identity and concentrations of these other adjuvants is largely unknown because commercial formulations are proprietary. This makes it difficult to parse out the exact causes of toxicity in these formulations as the individual components cannot be directly tested. Thus, while we can say with confidence that pure glyphosate has significantly lower toxicity to *D. japonica* than the two tested GBHs, more comparative studies evaluating the toxicity of glyphosate and various GBHs are necessary to better understand this toxidrome.

### 4.2 Glyphosate and GBHs do not cause toxicity to *Dugesia japonica* planarians at environmentally relevant concentrations

Both GBH formulations had a lethality LOEL of 316 µM (53.4 mg/L) and behavioral LOELs of 31.6 µM (5.34 mg/L) and 100 µM (16.9 mg/L, RC and RR, respectively) in adult *D. japonica* planarians. These results agree with previously published toxicity values for 30% glyphosate isopropyl amine salt in *D. japonica*, which found a 96 h LC_50_ of 128 mg/L glyphosate ae, though exposure conditions differed (bulk exposure with daily renewal). A related planarian species, *G. tigrina*, was found to be more sensitive to glyphosate/GBH exposure with a 48 h LC_50_ of 36 mg/L (Córdova López et al, 2019), though behavioral and regeneration effects following 96 h exposure were seen at similar concentrations (starting at 3.75 mg/L). Notably, a different GBH formulation (Roundup^®^ Original), which does not contain any additional active ingredients but lists ethoxylated tallowamines as an inert ingredient, was used in the *G. tigrina* study. Here, we observed increased sensitivity of both *G. tigrina* and *D. dorotocephala* planarians compared to *D. japonica* to the same GBHs. This emphasizes that different planarian species can have different sensitivities to chemicals, as also reported in (Van Huizen et al, 2017; Ireland et al, 2020). Thus, care needs to be taken when making comparisons across species—even with the same chemical.

While this and previous studies have shown that different GBHs pose a toxic hazard to freshwater planarians, to understand whether this is of ecotoxicological concern requires consideration of environmental concentrations. Glyphosate is highly absorbed in the soil, where it is degraded by microorganisms (Matozzo et al, 2020). The rate of degradation depends on several environmental factors (Lacroix and Kurrasch, 2023), with different reports citing 50% dissipation rates ranging from 1.2—197.3 days (Saunders and Pezeshki, 2015). Because of this rapid degradation, glyphosate is generally found at low concentrations in the terrestrial and aquatic environment (Solomon, 2016; Matozzo et al, 2020). Reviews of studies from various aquatic ecosystems across the world reported water levels generally on the order of a few µg/L (Matozzo et al, 2020; Saunders and Pezeshki, 2015) but as high as a few mg/L in several studies (Edwards et al, 1980; Tzaskos et al, 2012; Avigliano and Schenone, 2015; Zhang et al, 2016). Thus, although overall environmental glyphosate levels appear to be low, because of differences in agricultural practices, soil properties and rainfall, there is the potential for contamination hot spots that may be of toxicological concern (Maggi et al, 2020). Therefore, from an ecotoxicological point of view, there appears to be a sufficiently protective margin of safety for GBH toxicity to several planarian species under normal environmental conditions. From a human neurotoxicity/developmental neurotoxicity standpoint, comparison to the maximum contaminant level for glyphosate in the U.S. (0.7 mg/L) may indicate potential risk, given that the goal is to protect the most vulnerable populations. Thus, generally a factor of 100 is used when extrapolating from animal models to humans (Konietzka et al, 2014). Moreover, exposure levels would likely be even greater for occupational workers that directly handle GBHs.

### 4.3 Regenerating planarians are less sensitive than adult planarians to the GBHs

Across all endpoints, regenerating planarians showed less sensitivity to the compounds than adult planarians. For RC, most of the same endpoints were affected at both developmental stages, but with effects occurring at higher concentrations in the regenerating planarians. The toxicological profile of regenerating planarians exposed to 316 µM RC suggests overt systemic toxicity as these planarians had abnormal morphology and were practically immobile. Thus, although this concentration was not significantly lethal in regenerating planarians, it is likely that lethality would have manifested with longer exposure. On the other hand, 316 µM RR caused significant lethality in both developmental stages, but with greater magnitude in adult planarians, while mild sublethal effects were only observed in adult planarians.

While known developmentally neurotoxic compounds, such as the organophosphorus pesticide chlorpyrifos, show increased potency in regenerating planarians compared to adult planarians (Zhang et al, 2019), here, none of the GBHs showed developmental selectivity when comparing nominal concentrations. Notably, it is possible that the *in vivo* concentrations differ between the two planarian developmental stages due to metabolic or pharmacokinetic differences between the two developmental stages, as previously discussed (Ireland et al, 2022). This could explain the apparent differences in sensitivity when only comparing nominal concentrations.

### 4.4 Trends found in *Dugesia japonica* agree with results in other non-mammalian organismal models

When compared to chronic exposure studies with other popular non-mammalian organismal models, such as developing zebrafish, nematodes, or frogs, we found that the general trends were conserved. For example, studies in developing zebrafish (de Brito Rodrigues et al, 2019), nematodes (Jacques et al, 2019), or frogs (Turhan et al, 2020; Howe et al, 2004) that had direct comparisons found that GBHs (of various formulations) were more toxic than pure glyphosate. Moreover, similar to the results reported here in planarians, non-lethality effects with pure glyphosate were often only seen at concentrations that also induced significant lethality (Sulukan et al, 2017; García-Espiñeira et al, 2018; Gaur and Bhargava, 2019; Lu et al, 2022). A few sublethal developmental effects, such as malformations (Zhang et al, 2017) and premature hatching (Fiorino et al, 2018), have been observed in some developing zebrafish studies, though in other studies these same phenotypes were either not present or only at lethal concentrations (Sulukan et al, 2017; de Brito Rodrigues et al, 2019; Gaur and Bhargava, 2019). Sublethal behavioral effects on head thrashing have also been observed after 24 h glyphosate exposure in nematodes (Wang et al, 2017)**.**

Our data with a day 12 lethality LOEL of 169 mg/L ae glyphosate show good agreement with a previous study on *D. japonica* that reported a day 4 LC_50_ of 128 mg/L ae when using the glyphosate isopropyl amine salt (Zhang et al, 2023). However, when compared to other models, we found that intraspecies potency of pure glyphosate differed several orders of magnitude across published studies. For example, LOELs for lethality after 96 h varied from 0.05 to 400 mg/L ae in developing zebrafish (Sulukan et al, 2017; Zhang et al, 2017; de Brito Rodrigues et al, 2019; Gaur and Bhargava, 2019) and from 18.5 to 11840 mg/L ae in nematodes (Wang et al, 2017; Jacques et al, 2019). Differences in experimental methods and analysis can explain some of these discrepancies. These differences are even greater if studies with various GBHs are also considered. Given these vast differences in results, more studies into GBH toxicity are warranted, especially comparative studies testing many GBHs in parallel in the same system, to minimize confounding factors.

### 4.5 *Dugesia japonica* is a well-suited model for rapid testing of neurotoxicity and ecotoxicity

To perform large-scale comparative studies of many GBHs (and their individual components), inexpensive HTS methods that balance sensitivity and specificity are indispensable. While sensitivity is important to ensure potential hazards are identified - especially for a first-tier rapid screening model - if a system is overly sensitive, it cannot fulfill its purpose of decreasing the number of compounds that are being moved to the next tier of testing (Plunkett et al, 2010). As shown here, HTS in *D. japonica* was able to recapitulate the major findings observed with other common test systems, including human cell culture, and with comparable sensitivity. This suggests that planarians are a relevant and suitable system for evaluating glyphosate/GBH toxicity. The high-throughput capabilities of this system (Zhang et al, 2019) allow for large-scale comparative studies of a class of compounds. For example, planarian HTS and behavioral barcoding has been used to compare the phenotypic profiles of organophosphorus pesticides and connect specific behavioral effects with putative mechanisms (Ireland et al, 2022). In addition to their use as a biomonitor for ecotoxicity (Wu and Li, 2018), planarians are relevant for studying potential human health effects because of the complexity and highly conserved nature of the planarian nervous system (Ireland and Collins, 2023). Finally, planarians provide the unique capability to study both adult and developing brains in parallel using the same HTS assays, which allows for the distinction of neurotoxic from developmental neurotoxic effects. Together, these strengths make them a powerful model for studying GBH toxicity.

## Data Availability

The original contributions presented in the study are included in the article/Supplementary Material, further inquiries can be directed to the corresponding author.

## Supplementary material

Supplementary Data Sheet S1 Supplementary Figures and Tables.

Supplementary Data Sheet S2 Compiled data for each endpoint and chemical.
