# Supplementary material for: Comparative toxicity assessment of glyphosate and two commercial formulations in the planarian Dugesia japonica
Source: Front Toxicol. 2023 Jun 26;5:1200881. doi: 10.3389/ftox.2023.1200881 (PMC10332155; doi:10.3389/ftox.2023.1200881)
Supplement: Supplementary file 1 [file DataSheet1.pdf]

Supplementary Material

1 Supplementary Figures

(A)

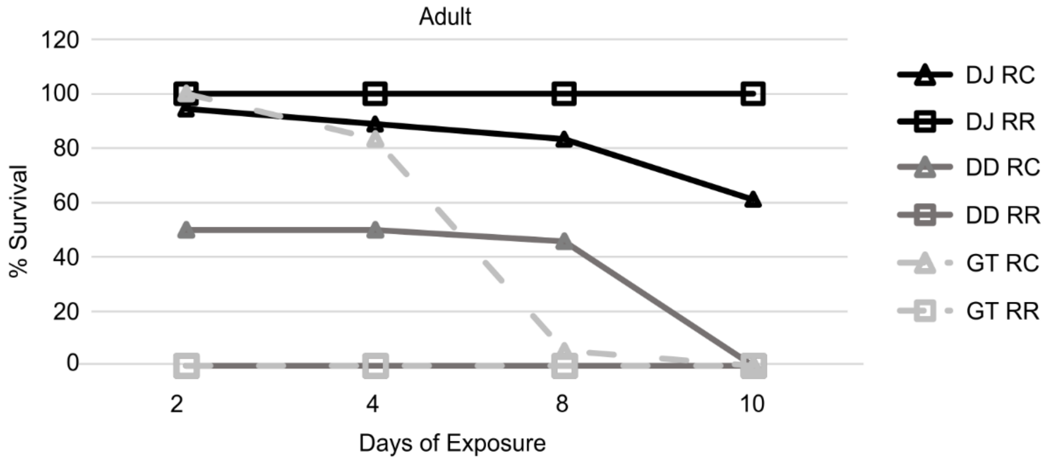

(B)

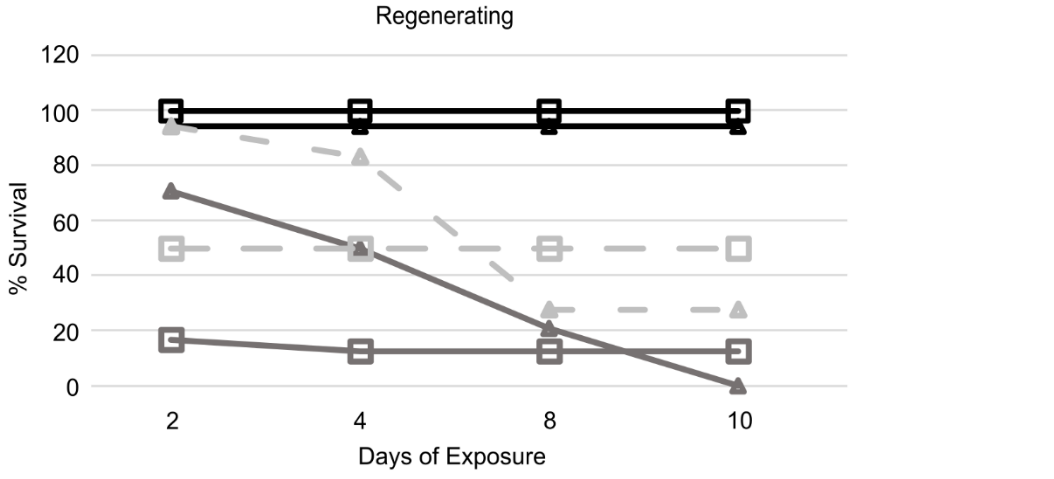

(C)

	Adult			Regenerating		
	DJ:DD	DJ:GT	DD:GT	DJ:DD	DJ:GT	DD:GT
RC_d2	**3.55E-03**	1	**9.46E-04**	0.16	1	0.16
RC_d4	**0.03**	1	0.07	**7.10E-03**	0.60	0.07
RC_d8	**0.02**	**1.22E-05**	**0.01**	**4.74E-06**	**1.24E-04**	0.72
RC_d10	**2.23E-05**	**1.59E-04**	1	**2.12E-10**	**1.24E-04**	**0.01**
RR_d2	**8.48E-12**	**3.31E-10**	1	**6.37E-08**	**4.45E-05**	0.28
RR_d4	**8.48E-12**	**3.31E-10**	1	**2.26E-08**	**4.45E-05**	0.14
RR_d8	**8.48E-12**	**3.31E-10**	1	**2.26E-08**	**4.45E-05**	0.14
RR_d10	**8.48E-12**	**3.31E-10**	1	**2.26E-08**	**4.45E-05**	0.14

Supplementary Figure S1. Comparison of lethality in adult and regenerating *D. japonica, D. dorotocephala* and *G. tigrina* planarians. (A-B) Percent survival over time of **(A)** adult planarians and **(B)** regenerating planarians in 316 µM RC or RR. Planarians were in bulk exposure conditions with 6 planarians per well, tested over 3-4 wells. Black coloring indicates *D. japonica* (DJ), dark gray coloring indicates *D. dorotocephala* (DD), and dashed light gray coloring indicates *G. tigrina* (GT).

(C) P-values for pairwise comparisons of lethality at 316 µM RC or RR in the different species for each worm type and day. Comparisons made using Fisher's Exact test with Benjamini and Hochberg correction for multiple testing.

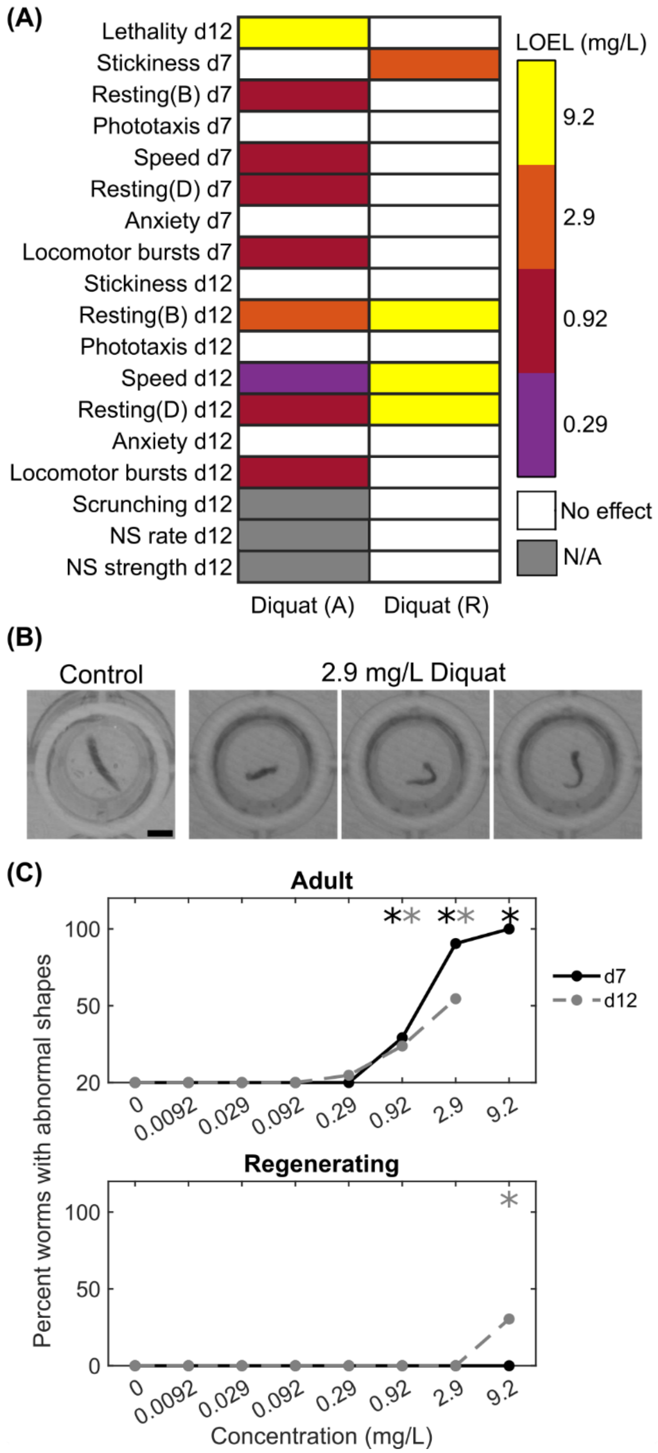

Supplementary Figure S2. Toxicity of diquat dibromide. (A) Heatmap showing the lowest observed effect level (LOEL) for diquat dibromide in adult (A) and regenerating (R) planarians in all tested endpoints. Percent time resting was calculated in both the dark (D) and blue (B) light periods of the phototaxis assay. NS: Noxious stimuli. For simplicity, only the results from stickiness (Z) are shown as no significant hits were found in stickiness (A). Only concentration-dependent hits are shown. The noxious stimuli assay could not be analyzed (N/A) for adult planarians due to a technical malfunction where the plate heated up faster than designed. All compiled data can be found in Supplementary File

S1. **(B)** Example images of a normal control planarian and the abnormal body shapes observed with 2.9 mg/L diquat dibromide in adult planarians. The diquat images are a time series of a single planarian every 0.5 sec to show the dynamic nature of the shapes. Scale bar: 2 mm. **(C)** Concentration-response curves for adult (top) and regenerating (bottom) planarians exposed to diquat dibromide. The percent planarians with any abnormal body shape was evaluated on both day 7 (d7, black solid line) and day 12 (d12, gray dashed line). Diquat at 9.2 mg/L was lethal to adult planarians on day 12 and thus no data are shown for adult planarians at this concentration on day 12. * $p<0.05$ using a Fisher's exact test, corrected using the Benjamini and Hochberg correction for multiple testing, comparing to the respective in-plate control population (see Supplementary Table S4).

2 Supplementary Tables

Supplementary Table S1. Overview of statistical methods.

Endpoint	Omnibus test	Post hoc test	Tails
Lethality	Fisher's exact (BH correction)*	N/A	Greater
Stickiness	Fisher's exact (BH correction)	N/A	Greater
Phototaxis	Fisher's exact (BH correction)	N/A	Less
Scrunching	Fisher's exact (BH correction)	N/A	Less
Speed	Welch's ANOVA	Tamhane-Dunn	Less
Locomotor bursts	Welch's ANOVA	Tamhane-Dunn	Both sides
Noxious Stimuli Rate	Welch's ANOVA	Tamhane-Dunn	Both sides
Noxious Stimuli Strength	Welch's ANOVA	Tamhane-Dunn	Both sides
Resting (dark and blue)	Kruskal Wallis	Dunn (BH correction)	Greater
Anxiety	Kruskal Wallis	Dunn (BH correction)	Greater

* BH: Benjamini and Hochberg correction for multiple testing

Supplementary Table S2. P-values for different term interactions for lethality in the bulk versus 96-well comparisons. Response refers to frequency of lethality, conc is chemical concentration, type is worm type (adult or regenerating), exp is exposure type (bulk or 96-well).

	**Gly_d7**	**RC_d7**	**RR_d7**	**Gly_d12**	**RC_d12**	**RR_d12**
response:conc:type:exp	1.00	1.00	1.00	1.00	NA	NA
response:conc:type	1.00	**9.54E-05**	1.00	0.94	0.85	0.11
response:conc:exp	1.00	**1.60E-03**	1.00	0.94	**0.02**	1.00
response:type:exp	**7.98E-04**	0.38	1.00	**1.01E-03**	0.74	1.00
conc:type:exp	0.07	0.81	0.90	0.28	0.92	0.92

Supplementary Table S3. P-values for different term interactions for GBH lethality in the species comparisons. Response refers to frequency of lethality, conc is chemical concentration, type is worm type (adult or regenerating), species refers to *D. japonica*, *D. dorotocephala*, or *G. tigrina*.

	**RC_d2**	**RC_d4**	**RC_d8**	**RC_d10**	**RR_d2**	**RR_d4**	**RR_d8**	**RR_d10**
response:conc:type:species	1	NA	NA	NA	1	1	1	1
response:conc:type	1	0.14	0.15	0.12	1	1	1	**3.64E-04**
response:conc:species	1	0.018	0.77	0.052	1	1	1	2.36E-01
response:type:species	0.294	0.85	**0.018**	0.63	1	1	1	**5.74E-05**
conc:type:species	0.97	0.99	0.36	0.44	**0.031**	0.063	0.075	**2.12E-04**

Supplementary Table S4. P-values for abnormal body shapes in diquat dibromide-exposed planarians. Fisher's exact test were performed against the respective in plate control populations. P-values were adjusted using the Benjamini and Hochberg correction for multiple testing. Concentration is in mg/L.

**Conc**	**0.0092**	**0.029**	**0.092**	**0.29**	**0.92**	**2.9**	**9.2**
Adult day 7	1	1	1	1	**0.0047**	**3.68E-09**	**1.01E-11**
Adult day 12	1	1	1	0.488	**0.0211**	**3.07E-05**	NA
Regen day 7	1	1	1	1	1	1	1
Regen day 12	1	1	1	1	1	1	**0.0039**
